# Editorial: Psychosocial rehabilitation for cancer patients

**DOI:** 10.3389/fpsyg.2023.1265258

**Published:** 2023-10-06

**Authors:** Xu Tian, Maria F. Jimenez-Herrera

**Affiliations:** ^1^Chongqing Traditional Chinese Medicine Hospital, Chongqing, China; ^2^Department of Nursing, Universitat Rovira i Virgili, Tarragona, Spain

**Keywords:** cancer, psychosocial rehabilitation, psychosocial issue, psychosocial mechanisms, psychosocial intervention

## Introduction

Cancer has become a major public health issue, as it leads to increased mortality and disability worldwide (Cheatley et al., 2021; Huang et al., 2023). In 2020, there were almost 19.3 million new cancer cases around the world (Sung et al., 2021); however, recent years have seen a rapid rise in the number of cured cancer patients (Miller et al., 2022), which leads to a concomitant upsurge in rehabilitation efforts (Weis, 2003). Rehabilitation is defined as “a process aimed at enabling persons with disabilities to reach and maintain their optimal functional capacity…”, while cancer rehabilitation aims at enabling cancer patients to restore their optimal physical, sensory, intellectual, psychiatric, psychological, and/or social function levels (Mikkelsen et al., 2008). Furthermore, psychosocial rehabilitation of cancer patients focuses on how to restore the psychosocial function of cancer populations. It has been proposed that cancer psychosocial rehabilitation should be an integral part of the care plan and that it should enjoy a status equal to that of surgery, radiation, and chemotherapy (Cheville, 2005). The current Research Topic entitled “*Psychosocial rehabilitation for cancer patients*” examined various facets related to the psychosocial rehabilitation for cancer patients, including psychosocial issues of cancer patients, conceptualization of psychosocial issues, early detection of psychosocial issues, potential mechanisms of psychosocial issues, and effective intervention for psychosocial issues.

## Psychosocial issues of cancer patients

Due to various reasons, such as cancer diagnosis and subsequent anticancer treatment, most cancer patients may suffer from various psychosocial issues (Lindert et al., 2021), such as psychological distress (Tian et al., 2021a) and social isolation (Liang et al.). For example, endocrine therapy is a standard treatment for hormone positive (HR+) breast cancer (BC) patients (Noordhoek et al., 2021), but the study by Zhao et al. revealed that side effects associated with this treatment significantly increased the risk of anxiety and depression. Psychosocial issues have been confirmed to be associated with increased risk of various adverse consequences, such as interruption of anticancer treatment, prolonged hospitalization stay, poorer quality of life (QoL), and increased mortality (Lindert et al., 2021). A preclinical study even showed that psychological stress can accelerate the growth of tumor cells (Zhang et al., 2020), and Zhang et al. found that psychological distress might increase the risk of incident lung cancer. Undoubtedly, psychosocial issues have become a major challenge in cancer setting given the associated increased risk of adverse consequences caused by these issues (Hyde et al., 2016).

Many efforts have been made to investigate how to effectively manage psychosocial issues of cancer patients (Chambers et al., 2015), as can be seen from the bibliographic analysis performed by Ahmad et al.. In their study, Ahmad et al. analyzed the research productivity and trends in psycho-oncology between 1980 and 2021, and confirmed a significant increase in publications over the past 5 years, with an annual growth rate of 13.9%. Meanwhile, they found that research hotspots mainly include interventions in cancer patients in developed countries, which further emphasizes the importance of paying more attention to psychosocial issues of cancer patients in low-income countries.

## Conceptualization of psychosocial issues

Clear and precise conceptual systems is crucial for developing and enriching nursing knowledge (Tofthagen and Fagerstrøm, 2010), which also emphasize that conceptualizing psychosocial issues should be the first step in starting research on how to effectively manage various psychosocial issues in cancer settings. For example, social isolation is not an emerging psychosocial issue (Berkman and Syme, 1979); however, an oncology-specific multidimensional definition of social isolation is yet to be systematically clarified. Liang et al. therefore performed an evolutionary concept analysis to conceptualize social isolation in adult cancer care, and comprehensively documented the antecedents, attributes, and consequences of social isolation, which further provides a basis for developing multidimensional assessment tools and intervention protocols to alleviate social isolation in adults with cancer.

## Early detection of psychosocial issues

Psychosocial issues can occur at any stage of cancer patients, and even throughout the entire disease course (Chambers et al., 2013). Early detection of psychosocial issues is the prerequisite of effective intervention; therefore, the development of appropriate tools is critically important (Sharpe et al., 2004; Kadan-Lottick et al., 2005). For example, there is currently no specific tool for lung cancer patients who may be at the high risk of suffering from psychological distress. Tian et al. (2021b, 2023) therefore developed an easy-to-use predictive algorithm to help identifying lung cancer patients at high risk of psychological distress in their subsequent study. Psychological flexibility (PF), an emerging concept in clinical psychology, refers to the ability to stay in contact with the present moment and pursue behavioral goals based on personal values and situational contexts (Cherry et al., 2021). Although many tools are available for measuring PF in non-cancer patients, the PF of cancer patients may differ from that of non-cancer patients, therefore proposing the urgent need of developing a specific instrument to this psychosocial issue. Ou et al. developed the Cancer-related Psychological Flexibility Questionnaire (CPFQ), which helps to reliably measure psychological flexibility in cancer patients and thus facilitate intervention for high-risk patients. However, we also need to realize that the levels of PF may differ between patients with different types of cancer, therefore more studies are still needed to clarify various open problems.

## Potential mechanisms of psychosocial issues

We must recognize that elucidating the mechanisms of psychosocial issues is critically important for developing precise intervention protocols. Emotional self-care (Mesurado et al., 2018), psychological distress (Osmani et al., 2023), and fear of cancer recurrence (FCR) (Luigjes-Huizer et al., 2022) have been showed to be important psychological issues during anticancer treatment. However, due to the lack of elucidation of the underlying mechanisms of these issues, there is still controversy over how to accurately target these issues to improve anti-cancer effects. In this Research Topic, three studies did efforts to investigate the influencing factors of different psychosocial issues or elucidate the possible associations between different psychosocial variables related to a certain one of these three psychological issues. Sebastian et al. used qualitative method to identify eight influencing factors related to emotional self-care, and also explained how different factors influence emotional self-care practices. Tian, Tang et al. revealed that mindfulness may alleviate psychological distress of lung cancer patients by reducing the level of illness perception and perceived stress. Yu et al. reported that good social support can directly mitigate FCR among Chinese breast cancer survivors, while illness uncertainty can play a mediation role between social support and FCR. These three studies provide an evidence basis for developing intervention protocols, and also provide an optional methodological framework for elucidating the mechanisms of other psychosocial issues in cancer patients.

## Effective intervention for psychosocial issues

Effective intervention is crucial for managing cancer patients who may experience or be experiencing psychosocial issues. There are two ways to choose intervention protocols, one is to conduct evidence-based evaluation of existing interventions, and another is to develop new intervention protocols. From the perspective of feasibility and economy, the first one should be preferentially considered. Crow et al. systematically evaluated the role of psychosocial interventions in reducing cortisol among BC patients, and reported that certain types of psychosocial interventions reduce cortisol (indicator of chronic stress) in BC patients. These findings also indicate that different types of psychosocial interventions may have different potential in addressing different psychosocial issues in different cancer patients. Therefore, Tian, Yi et al. separately evaluated the effectiveness of the mindfulness-based stress reduction (MBSR) program on psychological states and QoL in lung cancer patients, and suggested that the MBSR approach should be recommended as a part of the rehabilitation program for lung cancer patients. However, under certain special conditions, such as COVID-19 pandemic, the second way may be better for effectively managing psychosocial issues. Notably, it is difficult to deliver MBSR program in in-person approach during COVID-19 pandemic. With the popularization of the Internet in the healthcare field (Oh et al., 2005), mixed intervention models targeting the psychosocial issues of cancer patients have been widely recognized (Peng et al., 2007). Chang et al. developed an internet-delivered MBSR (iMBSR) program, and also evidenced that iMBSR improve mental health, body image, and self-efficacy in BC patients. Baussard et al. found that, although both hypnosis and cognitive behavioral therapy have been applied in cancer settings, they are still understudied on the symptom of fatigue in the colorectal cancer (CRC), and such programs have never been evaluated in Europe. Therefore, these authors developed hypnosis and cognitive behavioral therapy with online sessions, and designed a prospective, single-center, randomized interventional feasibility study to evaluate the role of this program in reducing fatigue in patients undergoing chemotherapy for a metastatic CRC. It is reasonable to believe that, after the formal study accomplishment, these authors will let us know the barriers/facilitators to the implementation of the program and the relevance of the program to the patients, and will generate hypotheses for a randomized control trial.

## Conclusion

Undoubtedly, current Research Topic provides valuable insights into the psychosocial rehabilitation of cancer settings, but also reveal some knowledge gaps that require further investigation. First, with the advancement of artificial intelligence technology, conventional psychosocial issues screening tool should be transformed into intelligent models. In addition, the forms of intervention should also be updated, but we must emphasize the challenges of implementing eHealth on an individual, environmental and technical level (Schreiweis et al., 2019). Second, more studies are required to deeply elucidate the possible psychosocial mechanisms of the most common psychosocial issues to develop precise intervention protocols. Finally, although it's recommended to manage psychosocial issues of cancer patients through psychosocial interventions (Riba et al., 2019), the role of traditional Chinese medicine (TCM) in managing these issues cannot be ignored (Tan et al., 2022). However, to promote TCM intervention protocols in clinical practice, further efforts are needed to standardize TCM intervention protocol.

## Author contributions

XT: Writing—original draft, Writing—review and editing. MJ-H: Conceptualization, Supervision, Writing—review and editing.
